# Supplementary material for: Effects of Anti-Seizure Medication on Sleep Spindles and Slow Waves in Drug-Resistant Epilepsy
Source: Brain Sci. 2022 Sep 24;12(10):1288. doi: 10.3390/brainsci12101288 (PMC9599317; doi:10.3390/brainsci12101288)
Supplement: Supplementary file 1 [file brainsci-12-01288-s001.zip › brainsci-1870751-supplementary.pdf]

Supporting Information: Supplementary Tables and Figures

Effects of anti-seizure medication on sleep spindles and slow waves in drug-resistant epilepsy

Jennifer K. Roebber[1,2*], Penelope A. Lewis[1], Vincenzo Crunelli[3,], Miguel Navarrete[1], Khalid Hamandi[1,2*]

[1] Cardiff University Brain Research Imaging Centre (CUBRIC), School of Psychology, Cardiff University, Maindy Rd, Cardiff CF24 4HQ, UK;
[2] The Welsh Epilepsy Unit, Department of Neurology, University Hospital of Wales, Heath Park, Cardiff, UK. CF14 4XN
[3] Neuroscience Division, School of Bioscience, Cardiff University, Cardiff CF10 3AX, UK;

* Correspondence: jroebber@gmail.com (J.K.R.);

Table S1: Individual clinical characteristics

Age	Sex	Epilepsy type	MRI	Interictal EEG	Ictal EEG	ASMs	Seizures (no ASM)	Seizures (ASM)
19	M	TLE	L HS	Sh and SW, occ R SW	No seizures recorded	LTG, ZNM	0	0
20	F	TLE	R HS	Occ R T Sh	No seizures recorded	LTG, PMP	0	0
21	M	FLE	Normal	Normal	Movement artefact only*	LEV, PMP, VPA	0	1
22	F	TLE	L T FCD	L T slowing	L T rhythmic theta	CLN, LTG	5	1
26	F	TLE	R T DNET	L T slowing	L T rhythmic theta	ESL	0	0
35	F	OLE	R O DNET + L T PMG	L T Sh	No seizures recorded	GBP, LCM	1	0
37	F	FLE	R F FCD	R T slowing	Attenuation, then R Delta	ESL	0	0
39	M	FLE	L F Lesion	Normal	L FC evolving slow rhythm	CLB, LEV, VPA, ZNM	0	0
40	M	TLE	R HS	R T Slowing	No seizures recorded	CLB, LEV, PGB, VPA	0	0
43	F	TLE	L T FCD	L T Slowing	L T rhythmic theta	LTG	0	0
44	M	TLE	L T cavernoma	L T Sh	L T rhythmic theta/delta	LTG, LEV, PMP	2	0
47	F	TLE	L HS	L T Sh	No seizures recorded	LTG	0	0
47	M	PLE	R F encephalomalacia	Normal	No seizures recorded	LEV, VPA, TPM	0	0
49	M	TLE	Normal	L T Sh	No seizures recorded	ESL, LEV, ZNM	0	0
60	F	TLE	R HS	L T slowing	No seizures recorded	LEV, VPA	0	0

Age	Sex	Epilepsy type	MRI Brain /CT Head	Video EEG	ASMs	Convulsive Events (Night 1)	Convulsive Events (Night 2)
20	F	DS	Normal	7 DS, normal EEG	TPM	1	1
21	F	DS	Normal	2 DS, normal EEG	LEV	0	0
23	M	DS	Normal	No events, normal EEG	VPA	0	0
26	F	DS	Normal	3 DS, normal EEG	GBP, LTG, LEV	0	0
27	F	DS	Normal	2 DS, normal EEG	DZP	0	0
32	F	DS	Normal	3 DS, normal EEG		0	0
32	F	DS	Normal	1 DS, normal EEG	LEV	0	0
37	F	DS	Normal	Multiple jerks, normal EEG		0	0
41	F	DS	Normal	1 DS, normal EEG	CLB	0	0
46	F	DS	L Parietal surgical cavity Resection of ganglioglioma	2 DS, normal EEG	ZNM	0	0
47	F	DS	Normal	7 DS, normal EEG	DZP, GBP, LTG	0	1
49	M	DS	Normal	2 DS, normal EEG	CBZ, PGB	0	0
54	F	DS	Normal	2 DS, normal EEg	VPA	0	0
65	F	DS	Normal	2 DS, normal EEG	VPA	0	1

TLE = temporal lobe epilepsy, FLE = frontal lobe epilepsy, OLE = occipital lobe epilepsy L = left, R = right, T = temporal, Sh = sharp wave, HS = hippocampal sclerosis, SW = slow wave, FCD = focal cortical dysplasia, DNET = dysembryoplastic neuroepithelial tumour, O = occipital. DS = Dissociative Seizures. *frontal hypermotor semiology.

Table S2: Demographics and sleep architecture

	Epilepsy database		Within group (epilepsy)	Control (DS) database		Within group (control)	Between group (epilepsy vs. control)
Number of patients	15			14			
Female	8			12			
Mean Age (years)	36.4			37.1			
Benzodiazepine users	2.0			3.0			
Sleep Architecture	No AED	Full AED	p value	Night 1	Night 2	p value	p value
Total sleep time (h)	4.8	5.2	0.3	6.6	6.9	0.5	**0.00004**
Stage 1 sleep (%)	6.8	3.2	**0.01**	4.7	3.1	0.1	0.3
Stage 2 sleep (%)	39.3	42.5	0.4	50.4	51.0	0.8	**0.02**
Slow wave sleep (%)	46.6	45.0	0.8	39.8	39.3	0.9	0.1
REM sleep (%)	27.3	29.2	0.5	25.1	26.6	0.5	0.4
REM/NREM Ratio	0.31	0.33	0.71	0.3	0.3	0.7	0.5
Sleep Onset Time	01:23	00:53	0.15	23:36	23:17	0.50	**0.00036**
Time of wake	7:20	7:10	0.57	7:34	7:20	0.47	0.58

Supplementary Table S2: Demographics and sleep architecture. The Epilepsy group showed higher time spent in N1 during the first recorded night, but not the Control group (Epilepsy: p = 0.01, DS: p=0.05). Total sleep time differed significantly (p=0.00004) between the two groups. Once normalized, the Control group spent more time in stage 2 (p=0.02), compared to those in the Epilepsy group. Time spent in stage 1, 3 and REM did not differ between groups (p=0.24, 0.16 and 0.43, respectively). Significance determined with paired student's t-test (within group) or unpaired for between group analysis, unequal variances were analysed with Welch's *t*-test.

Figure S1: power with or without benzodiazepines

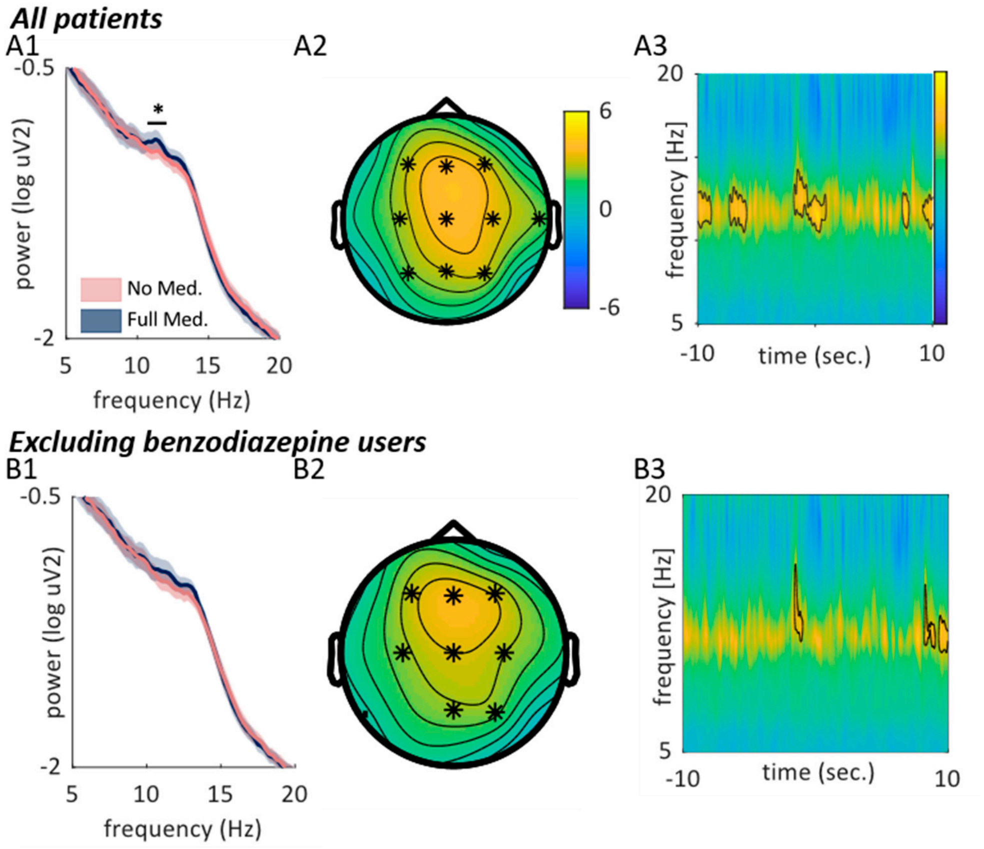

Supplemental Figure S1: Spindle power is enhanced with anti-seizure medication even when benzodiazepine users are removed. A1,B1) As in Figure 2, EEG power from 5-20Hz across frontal electrodes for stage N2 and N3 sleep in the Epilepsy group **A1**) with benzodiazepine users or **B1**) excluding benzodiazepine users, **A2,B2**). Topographic head plot of EEG power differences in spindle band (11-14hz) between medicated and unmedicated state in patients with epilepsy. Cluster based permutation tests reveal a significant difference across frontal, central, and parietal electrodes (**A2:** Cluster based permutation test: 10,000 iterations, cluster alpha threshold 0.06, **B2**; p=0.0060 p=0.014). **A3,B3**) Time frequency of power differences in detected spindles on Cz (time zero). A cluster-based permutation test reveals temporally spaced clusters after medication rather than nonspecific power increase in the spindle band (**A3:** 1,000 iterations, clusters highlighted with black contours, 6 positive clusters p=0.004-0.047, **B3**: p= 0.033-0.036, 3 positive clusters).

Figure S2: Spindles in (topological x frequency) space

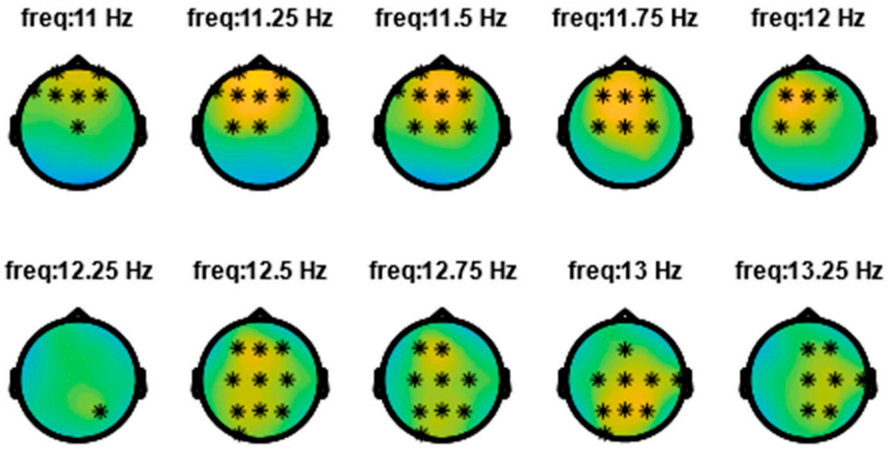

Supplemental Figure S2: Spindles in (topological x frequency) space: Cluster based permutation test of medication across the entire spindle range (10-16hz) in 0.25 Hz steps reveals two significant clusters with distinct topological profiles. A) The first significant cluster (p=0.033, frequency 11-12 Hz) centres over Fz. B) The second significant cluster (p=0.05, frequency 12.25-13.25 Hz) centres around Cz. Both clusters show an increase in power with medication. These are the topologically expected locations for fast and slow spindles. Both groups showed an increase in power with medication, so the broader 11-16 Hz spindle band was used for the main analysis.

Figure S3: Group level effects of ASMs on spindles

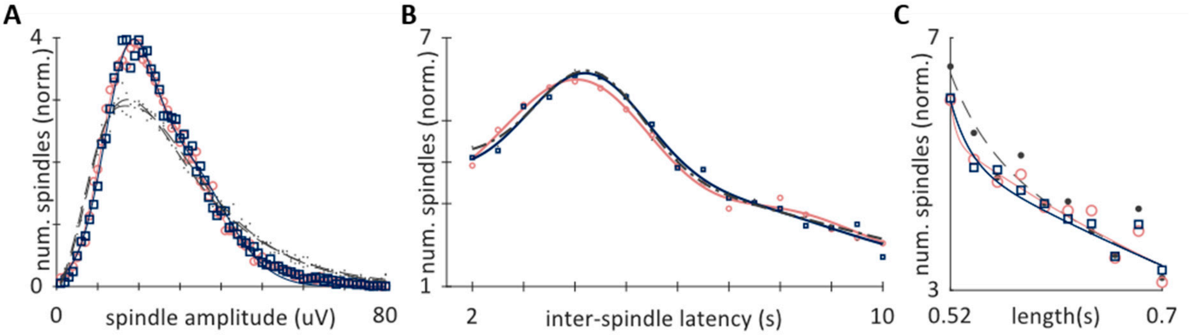

Supplemental Figure S3: Group level effects of ASMs on spindles: These plots are group-level analyses of Figure 3. Pink shows the Epilepsy group on nights without medication, blue shows the Epilepsy group on nights with medication. Controls are shown in grey. **A)** Between group, the Epilepsy group showed significantly higher spindle amplitudes compared to the Control group (Two-Sample Kolmogorov-Smirnov Test, d=0.038, p=3.5x10-33 mean amplitude. This effect survived when benzodiazepine users were excluded (d=0.058, p=1.7x10-59). For both groups, the mean amplitude increased by 0.3uV on the second night (Epilepsy group: d=0.013, p=0.0037, Control group: d=0.013, p = 0.045). **B)** Inter-spindle intervals showed a significant left shift in the Epilepsy Group on nights without medication, compared to medicated nights or the Control group. That is, inter-spindle intervals were significantly increased with ASMs (two-sample Kolmogorov-Smirnov test, d= 0.014, p=0.038). The control group did not differ between night (d=0.017, p=0.069). There was no significant difference between groups (d=0.0043, p=0.95). **C)** Spindle length was slightly reduced between nights for both groups (two sample Kolmogorov-Smirnov test, Epilepsy: d=0.013, p=0.004. control: d=0.016, p=0.007) but did not differ between group (d=0.0080, p=0.08).

Figure S4: ASM effects collapsed across Epilepsy and Control groups and regressed against age

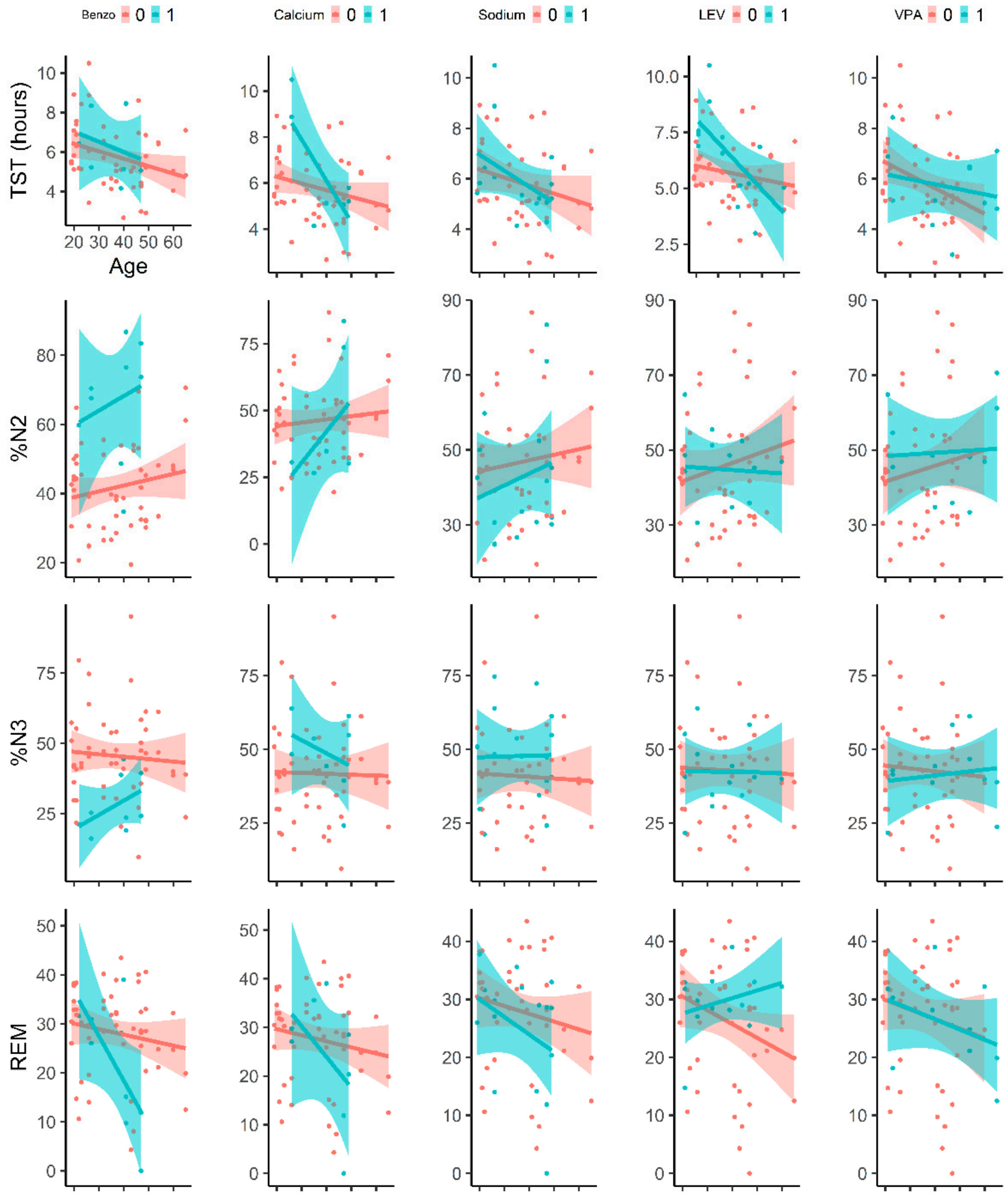

Supplemental Figure S4: ASM effects collapsed across Epilepsy and Control groups and regressed against age. Each column examines the impact of a specific ASM class on each sleep stage with the presence of the ASM (blue, “1”) compared to the absence (red, “0”). Rows show, TST = total sleep time; %N2 = relative percentage of stage 2 sleep; %N3 = relative percentage of stage 3 sleep. Data is collapsed across diagnosis (showing both those with Epilepsy and Controls). Linear mixed models for each condition were tested, with participant and ASM as fixed effects. ASMs significance was determined using Repeated Likelihood Ratio Tests and Akaike Information Criteria.

Figure S5: ASM effects on sleep parameters

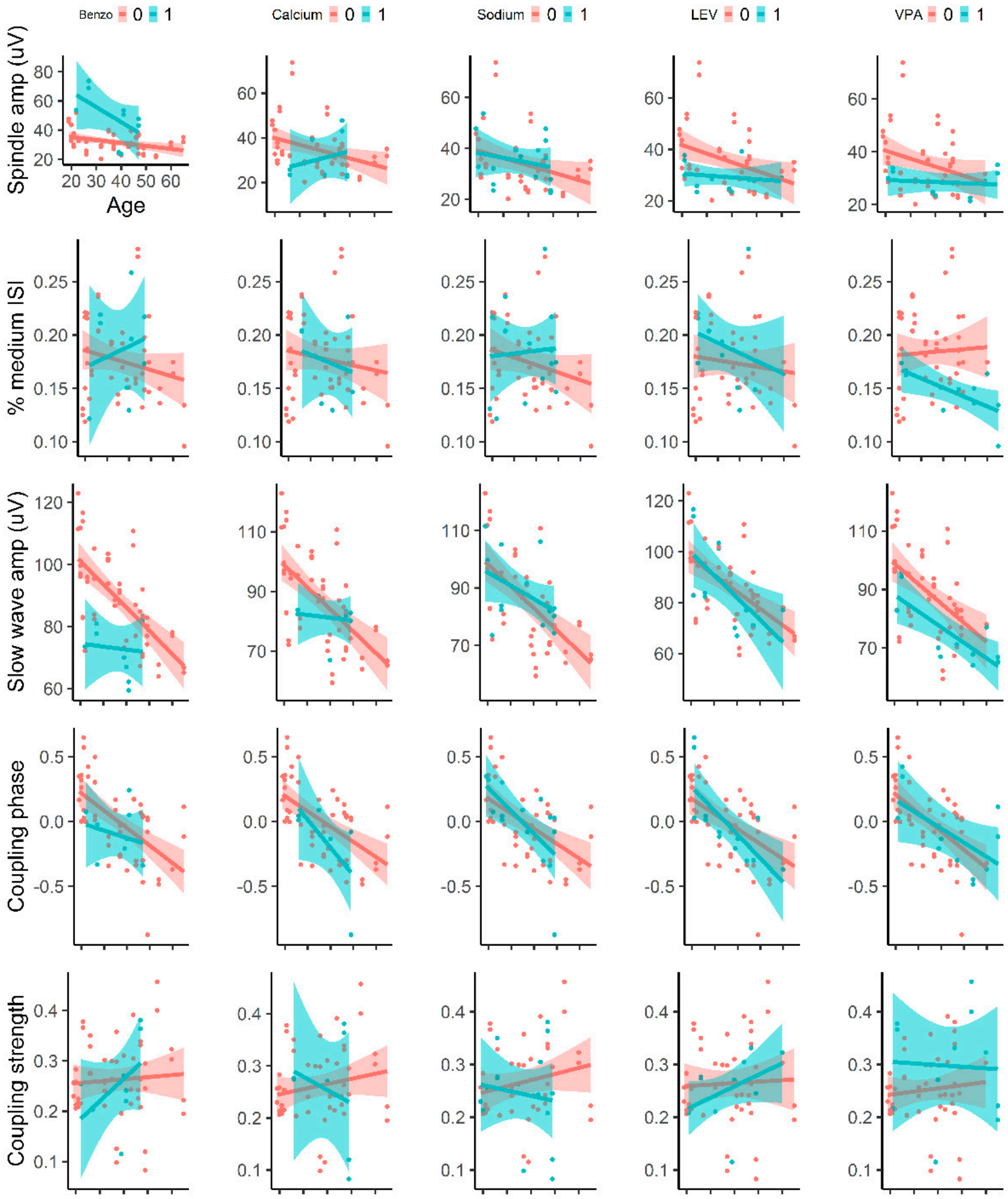

Supplemental Figure S5: ASM effects on sleep parameters: Each plot examines the impact of a specific ASM class on a sleep feature. For example, the top left regresses total sleep time (y-axis) against age (x-axis) with those taking the ASM depicted in blue ("1") according to the legend (column variable). In the top left, red points show participants not taking Benzodiazepines, while Blue points show those taking Benzodiazepines. Data is collapsed across diagnosis (showing both those with Epilepsy and Controls). Linear mixed models for each condition were tested, with participant and ASM as fixed effects. ASMs significance was determined using Repeated Likelihood Ratio Tests and Akaike Information Criteria.
